# A case report of seronegative cat scratch disease, emphasizing the histopathologic point of view

**DOI:** 10.1186/1746-1596-9-62

**Published:** 2014-03-19

**Authors:** Ok Ran Shin, Yang Ree Kim, Tae-hyun Ban, Taeseok Lim, Tae Hee Han, Su Yeon Kim, Kyung Jin Seo

**Affiliations:** 1 Department of Hospital Pathology, Uijeongbu St. Mary’s Hospital College of Medicine, The Catholic University of Korea 271, Cheonbo-ro, Uijeongbu-si, Gyenggi-do 480-717, Republic of Korea; 2 Department of Internal Medicine, The Catholic University of Korea, College of Medicine, Seoul, Korea; 3 Department of Laboratory Medicine, Inje University Sanggye Paik Hospital, Seoul, Korea; 4 Division of Zoonoses (CJD/Rabies/LB), Center for Immunology & Pathology, National Institute of Health (Korea CDC), Seoul, Korea

**Keywords:** Cat scratch disease, Bartonella, Warthin-Starry silver stain, PCR, Histological features, Differential diagnosis

## Abstract

**Abstract:**

Cat scratch disease, necrotizing granulomatous lymphadenitis caused by *Bartonella henselae,* usually benign and self-limited. However, various clinical manifestations and no pathognomonic histopathologic features can lead to misinterpretations and diagnostic disputes. We report a case of cat scratch disease in a 39-yr-old male patient with fever and left axillary lymphadenitis. He had a history of cat bite on the left hand dorsum. On excision, the lymph node showed follicular hyperplasia, stellate microabscesses with a rim of granulomatous inflammation. Warthin-Starry silver staining showed many clumps of silver-stained bacilli within the necrotic foci. Serological tests were negative. Diagnosis was established by PCR analysis.

**Virtual slides:**

The virtual slides for this article can be found here: http://www.diagnosticpathology.diagnomx.eu/vs/1877499238123059

## Letter to the editor

Cat scratch disease (CSD) is caused by *Bartonella hensela*e [1-3]. The pathogens are transmitted by bites or scratches from infected cats. Usually a unilateral lymphadenitis near the scratch, bite site develops 2–3 weeks after infection. CSD is a common cause of chronic lymph node swelling in children and adolescents [1-3]. Histopathologically, the affected lymph node shows granuloma with microabscess formation, which is not pathognomonic. We present a case of CSD, serologically negative but positive by Warthin-Starry (WS) stain and confirmed by PCR, discuss histopathologic features and review the literature, emphasizing histopathologic differential diagnosis.

A 39-year-old male patient was admitted with painful swelling in the left axilla and elbow and fever for 3 weeks. He had a history of cat bite on the left hand dorsum 3 weeks before admission. His temperature was 39.3°C. A rash was noted on the left hand dorsum. Two tender palpable lymph nodes were present at the left axillary and epitrochlear regions. Laboratory results were unremarkable. Liver enzymes were normal. Blood cultures and viral serologic tests results were all negative.

An indirect fluorescence assay (IFA, commercial kit) against *Bartonella (B.) quintana* and *B. henselae* gave negative results (IgG <1:16, IgM <1:16). Serologic tests for Tularemia and Toxoplasmosis were also negative. Although the fever subsided after 3 days of antibiotics, the axillary lymphadenopathy didn’t resolve and an excisional biopsy of the affected lymph nodes was performed.

**Figure 1:**
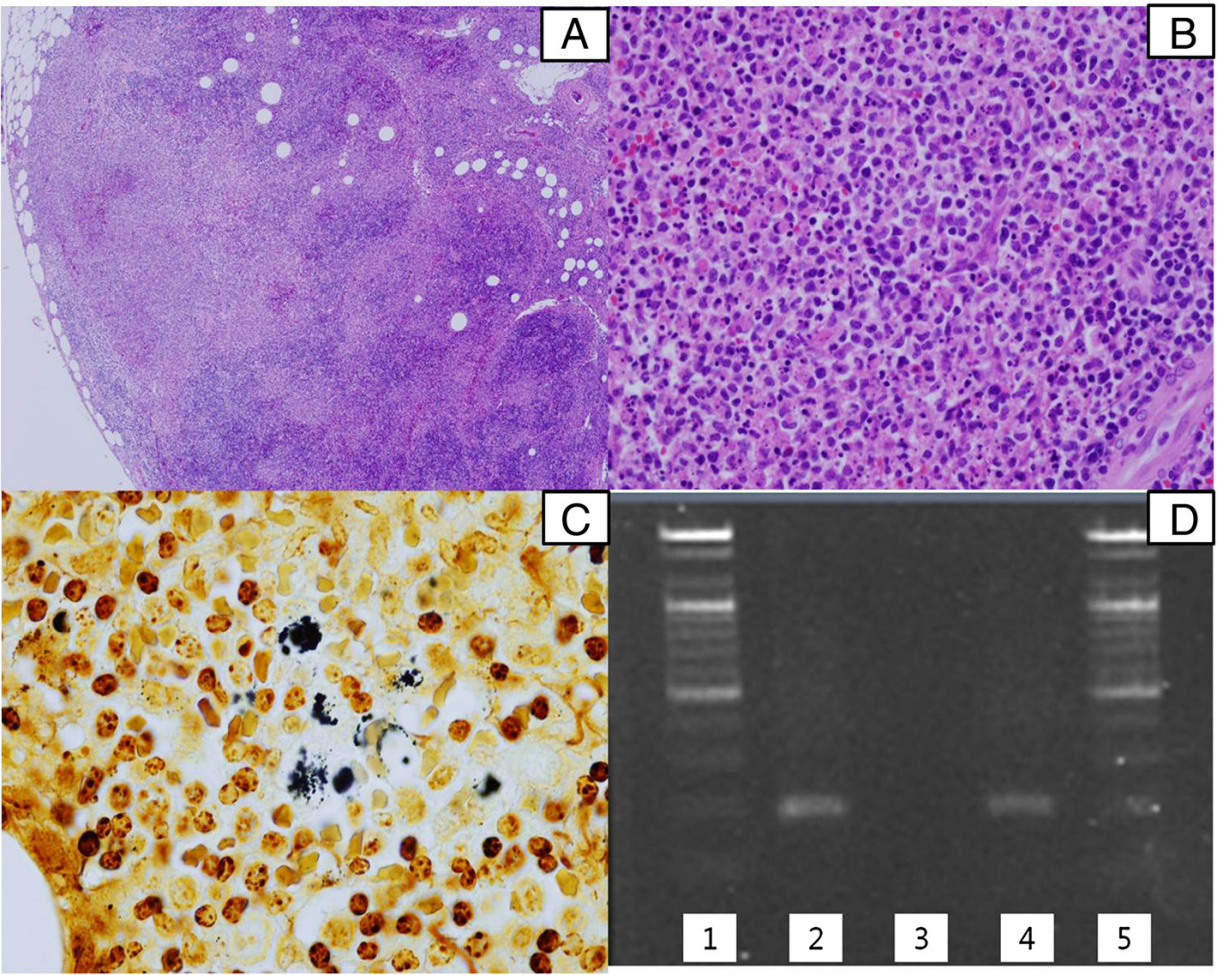
**Histopathology, silver stain and PCR of Cat scratch disease. (A)** The affected lymph node showed reactive follicular hyperplasia and multiple geographic microabscesses (H&E stain × 40). **(B)** Numerous neutrophils are seen in the necrotic foci of the lymph node (H&E stain × 400). **(C)** Some clumps of bacteria were found in the lymph node (Warthin-Starry silver stain × 1,000). **(D)** Results of semi-nested polymerase chain reaction (PCR) for the *B. henselae pap 31* gene (211 bp). From the left column, Lanes 1-5: Lane 1 and 5, DNA ladder marker (Bioneer, Daejeon, Korea); lane 2, positive control (Houston-1, ATCC 49882); lane 3, negative control; lane 4, lymph node tissue from the patient.

Pathologically, the lymph node demonstrated granulomatous inflammations with multiple stellate microabscesses with numerous neutrophils within the necrotic foci (Figure 1A & B). Culture results were negative for any other microbes. The Brown-Brenn Gram stain was negative. A PAS and a methenamine silver stain showed no fungal species, but the WS stain showed some clumps of silver-stained bacilli in areas of the necrosis (Figure 1C). The final diagnosis was established by PCR using formalin-fixed paraffin-embedded (FFPE) lymph node tissue. PCR was performed with the primer sets (PAPn1, PAPn2, and PAPns2) for the *pap31* gene, according to the previous description by Zeaiter et al. (Figure 1D) [4].

Ever since the first description by Robert Debre in 1931, the terminology of *Bartonella* species have experienced some changes [3,5-7]. Briefly, in 1993, *B. henselae* is recognized as the causative agent of CSD [8-13]. Most patients with CSD have cat exposures, i.e. scratches or bites [3,7,14]. The most typical form of CSD manifests as tender, regional lymphadenopathy 2 weeks after an inoculation. The results of routine lab studies are unremarkable [3,15]. Systemically, most patients might experience fever, generalized aches, malaise, anorexia, nausea, and abdominal pain [5,9].

As Florin et al. summarized, clinical manifestations of *B. henselae* are expanding, and the list includes typical CSD (fever and localized lymphadenopathy only), prolonged fever/fever of unknown origin and hepatosplenic disease, and less common various manifestations [9].

Culture is not reliable for the diagnosis of CSD, as the organism is slow-growing and difficult to culture [3,16]. More recently, Serology and PCR have been applied widely [9]. Despite high specificity (100% in 1 study), PCR shows a low sensitivity, ranging from 43% to 76% [9,17,18]. A more practical means is a serology for *B. henselae* antibodies [9,19]. One of the serologic test is an indirect fluorescence assay (IFA) [9]. In our case, serologic test, i.e. IFA gave negative results (IgG <1:16, IgM <1:16). However, PCR assay of FFPE lymph node tissue was positive.

Tissue biopsy is one useful way to diagnose CSD and to exclude other etiologies, despite its invasive nature [3,9]. Silver impregnation stains (Warthin-Starry (WS) or Steiner) are preferred to detect *Bartonella* organisms; however, organisms are variably detectable, and these stains are technically challenging [3].

The histologic differential diagnosis of CSD lymphadenitis primarily includes Kikuchi necrotizing lymphadenitis (KNL), Kawasaki disease and other infectious processes, such as tularemia, mycobacterial infection, brucellosis, fungal infection, lymphogranuloma venereum (LGV) and lymphadenitis associated with idiopathic granulomatous mastitis (especially when in a young female patient, though in this case, the patient was male) [1,3,20]. LGV mainly involves the inguinal lymph node. Serologic studies, special stains for *Bartonella,* and molecular studies might be required to distinguish between the two.

In our case, histopathologically, the lesion showed suppurative granulomatous inflammation, and the WS stain detected clumps of bacilli, highly suggestive of CSD but not definitive [21,22]. Chondrogiannis et al. described a sero-negative CSD case confirmed by PCR and mentioned limitations of histopathology [21]. However, they also mentioned histopathologic examination of lymph nodes is crucial for exclusion [21,23].

In our case, the serology for tularemia was negative. No microorganism was found by a Brown-Brenn Gram stain. No fungal species were detected by the PAS stain.

KNL can be histologically similar to CSD, however, KNL lacks neutrophils or epithelioid cells with granulomatous arrangements [24].

Ultimately, no single criterion can be considered the diagnostic gold standard [9]. In 2000, Margileth proposed a diagnostic criteria for *B.* infection (three of 4 of the following): 1. cat or flea contact regardless of presence of inoculation site, 2. negative serology for other causes of adenopathy, sterile pus aspirated from a node, a positive PCR assay, and/or liver/spleen lesions seen on CT scan, 3. positive enzyme immunoassay or IFA assay with a titer ratio of ≥1:64, and 4. biopsy showing granulomatous inflammation consistent with CSD or a positive WS stain [9,25].

We report a case of CSD, sero-negative but confirmed by PCR, with histopathologic features. Characteristic histopathologic findings combined with the WS stain, may contribute to the diagnosis.

## Consent

Written informed consent was obtained from the patient for publication of this Case Report and accompanying images. A copy of the written consent is available for review by the Editor-in-Chief of this journal.

## Abbreviations

CSD: Cat scratch disease; KNL: Kikuchi necrotizing lymphadenitis; IFA: Indirect fluorescence assay; PCR: Polymerase chain reaction; LGV: Lymphogranuloma venereum.

## Competing interests

The authors declare that they have no competing interests.

## Authors’ contributions

YRK, THB and TSL collected the clinical data. ORS made the final diagnosis of this disease. SYK performed serologic tests. THH performed PCR test. KJS and ORS drafted the manuscript. All authors have read and approved the final manuscript.
